# Leuprolide and triptorelin treatment in children with idiopathic central precocious puberty: an efficacy/tolerability comparison study

**DOI:** 10.3389/fped.2023.1170025

**Published:** 2023-05-17

**Authors:** M. Valenzise, C. Nasso, A. Scarfone, M. Rottura, G. Cafarella, G. Pallio, G. Visalli, E. Di Prima, E. Nasso, V. Squadrito, M. Wasniewska, P. Irrera, V. Arcoraci, F. Squadrito

**Affiliations:** ^1^ Department of Human Pathology and Evolutive Age “Gaetano Barresi”, University of Messina, Messina, Italy; ^2^ Department of Clinical and Experimental Medicine, University of Messina, Messina, Italy

**Keywords:** GnRHa, central precocious puberty, triptorelin, leuprolide, pediatric

## Abstract

**Introduction:**

Central precocious puberty (CPP) results from premature activation of hypothalamic-pituitary-gonadal axis, with the consequent increase of gonadotropin-releasing hormone (GnRH); GnRH agonists (GnRHa) represent the gold-standard therapy in children with CPP although their use might be responsible for pituitary GnRH receptors down-regulation, that in turn suppresses luteinizing hormone (LH) and follicle stimulating hormone (FSH) and blocks of gonadal sex hormones release. The most prescribed GnRHa in the clinical practice are leuprolide and triptorelin, whose use is generally safe and well tolerated; however, mild menopausal-like side effects could appear. The aim of the present study was to investigate and compare the efficacy and tolerability profile of leuprolide and triptorelin in CPP patients.

**Methods:**

110 girls affected by CPP were enrolled in this retrospective study, carried out from 2018 to 2020. The enrolled patients received leuprolide (*n* = 48) or triptorelin (*n* = 62). Efficacy was investigated by the means of clinical parameters and radiological changes and side effects were also recorded to evaluate the possible relationship between the two GnRHa treatments and side effects appearance.

**Results:**

At baseline triptorelin patients had significantly higher LH and LH peak levels than leuprolide patients, whereas no significant difference in other patient characteristics was observed between the two groups. The leuprolide treatment lasted 971 days [790–1,171 days] while the duration of triptorelin administration was 792 days [760–1,003 days] (*p* < 0.001). Overall 46 (41.8%) of the studied patients reported mild menopausal-like symptoms: among these 27 were treated with triptorelin and 19 with leuprolide (*p* = 0.558). Patients treated with triptorelin, or leuprolide showed headache (27.4% vs. 16.7%), mood swings (12.9% vs. 16.7%), increased appetite (12.9% vs. 18.8%) and nausea (1.6% vs. 10.4%) respectively. Moreover, the onset of side effects appearance related to GnRHa therapy significantly reduces with the increase of the initial bone age (*p* = 0.038).

**Conclusion:**

Leuprolide and triptorelin treatment appear to be effective and safe without significant difference between the two drugs in term of efficacy and tolerability, making both good options for treating CPP.

## 1. Introduction

Puberty represents a physical, hormonal, and psychological changeover period from childhood to adulthood which is influenced by genetic, nutritional, environmental and socioeconomic factors (1). This period is characterized by an increase of gonadotropin-releasing hormone (GnRH) release from hypothalamus (2, 3) that stimulates development of secondary sexual characteristics as well as maturation of the growth plates and increase in height velocity (4). Precocious puberty (PP) is clinically described as a premature pubertal development that occurs at Tanner stage B2 before the age of 8 years in girls and at Tanner stage G2 before the age of 9 years in boys. Central precocious puberty (CPP) refers to premature activation of hypothalamic-pituitary-gonadal (HPG) axis, with the consequent increase of pituitary luteinizing hormone (LH) release and reduction of follicle stimulating hormone (FSH) secretion (5). Usually, breast development (thelarche) represents the earliest clinical manifestation of CPP in girls, followed by pubic hair appearance (pubarche). The overall incidence of CPP is estimated to be approximately 0.05% and 0.2% in boys and girls, respectively (6). CPP can be idiopathic or secondary to various causes such as genetic syndromes, intracerebral tumors, and chronic exposure to sex steroid hormones. Idiopathic precocious puberty is the most common cause of CPP, mainly observed in girls (90%) (1, 3). Genetic factors, race, diet, and environmental changes could be involved in the pathogenesis of CPP (7–9). In the last years, among the environmental factors, COVID-19 pandemic caused millions of people to stay quarantined in their homes for a long time (10, 11) and this condition impaired the timing of puberty, as a consequence of overweight and psychological problems (12–14); in fact, after the lockdown period, an increase of CPP diagnosis was observed. Also, CPP may induce psychosocial problems, thus contributing to mental and physical consequences on pediatric patients and their parents, as well as financial burden for parents and the entire society (15). Girls affected by CPP may benefit from treatment with GnRH analogues to prevent premature fusion of epiphyses and preserve adult height potential. In this context, Gonadotropin-releasing hormone agonists (GnRHa) represent the standard of care for CPP treatment (9) and leuprolide and triptorelin are the most prescribed drugs (9, 16). Clinical trials and post-marketing pharmacovigilance surveillance suggest good tolerability and few mild menopausal-like side effects due to GnRHa treatment (17). In particular, drug tolerability refers to the degree of the adverse effects that can be tolerated by patients: it is of paramount importance because it may influence the adherence to therapy and compliance of patients. Therefore, the aim of this study was to investigate and compare the efficacy and tolerability profile of leuprolide and triptorelin in CPP patients.

## 2. Methods

### 2.1. Case collection

A retrospective observational study including 110 female patients with CPP was carried out in the University Hospital “G. Martino” of Messina (Sicily, Italy) between January 2018 and December 2020. The study was approved by the Ethics Committee of Messina Hospitals (n° prot 130/22 data of approval 13/12/2022), according to the legal requirements concerning observational studies. CPP was defined in accordance with the following criteria: breast development before the age of 8 years, Estradiol (E2) levels >20 pg/ml, LH/FSH >1 and uterine length >36 mm. The algorithm for the diagnosis of CPP was used based on basal LH level; particularly, basal LH level greater than 0.1 mUI/ml in girls with breast growing and physical sign of PP (e.g., accelerated growth rate and bone age maturation) was considered indicative of central puberty activation. The GnRH stimulation test was considered mandatory for the recognition of the HPG axis activation in girls with physical signs of PP but with basal LH level <0.1 mUI/ml (18). Patients with negative results in the GnRH stimulation test or with clinical conditions, such as neurological/neurosurgical and genetic disease, brain tumor, trauma, infection, hypothalamic-pituitary congenital malformations, and those suffering from non-idiopathic CPP were excluded from the study. Moreover, patients clinical-auxological, bio-humoral and instrumental data were evaluated. Specifically, the following clinical-auxological details were considered: chronological age, weight, height, predicted adult height (PAH), bone age and Tanner's stage (pubic hair and breast growth) (19) before and at the end of treatment. Bone age was calculated using radiographs of left hand and wrist according to the standards of Greulich and Pyle (20). All patients included in the study were at Tanner stage 2 according to breast appearance and at Tanner stage 1 according to pubic hair growth. Furthermore, all patients have a LH peak over than 5 mUI/ml or basal LH level greater than 0.1 mUI/ml with breast growing and physical sign of PP; therefore, all girls enrolled in the study can be considered affected by CPP. Moreover, bio-humoral evaluation including FSH and LH levels (both basal and post-stimulus with GnRH), somatomedine C (SMC) and E2 were measured at the beginning of treatment. In addition, the pelvic ultrasonography measures were carried out to calculate the ovarian volume, using the ovoid's formula (length × width × depth × 0.52). Patients were stratified according to the administered drug: both drugs were administered every 28 days by intramuscular injection at a dosage of 3.75 mg. Patients were treated until reached bone age maturation (attained when the epiphyseal plates close). Treated patients were asked through open-ended questions about any symptoms during follow-up every 6 months by telephone interview. All unfavorable and unintended symptoms were evaluated for severity, duration, seriousness and relation to the used drugs and outcome. We specifically checked all participants for mild menopausal-like side effects, including headache, increased appetite, mood disorders, nausea, pain at the injection site, and vomiting.

### 2.2. Statistical analysis

Descriptive and comparative analysis of patient characteristics treated with leuprolide or with triptorelin were carried out, both at baseline and at the end of treatment. In addition, descriptive and comparative analysis of the occurrence of side effects associated with leuprolide and triptorelin therapy were performed. Descriptive analyses were reported as absolute frequency and percentages or medians with interquartile range (IQR), for categorical and continuous variables, respectively. Since some of the numerical variables showed a not normal distribution (verified using the Kolmogorov–Smirnov test for normality), a non-parametric approach was adopted. The two-tailed Pearson chi-square test and the Mann–Whitney *U* test for independent sample were employed to compare categorical and continuous variables, respectively. Univariate logistic regression models were performed to identify predictors of side effects appearance. All variables identified as predictors were included in a stepwise multivariate logistic regression model (backward procedure, *α *= 5%). Moreover, all variables not resulted significant in the univariate analysis, but considered clinically remarkable after a careful consideration based on current knowledge and clinical expertise, and with a cut-off of alpha error of 0.2 according to Hosmer–Lemeshow test, were also included (21, 22). On the contrary, variables with the same clinically significant and with a plausible collinearity, verified by the Spearman's rank correlation coefficient, were excluded from the multivariate model. Odds ratios (ORs) with 95% confidence intervals (CIs) were calculated for each covariate of interest in univariate (crude OR) and multivariate (adjusted OR) regression models. The goodness of fit of the regression model was carried out by the Hosmer–Lemeshow test for adequacy. A *p*-value <0.05 was considered statistically significant. All statistical analyses were performed using SPSS version 29.0 (IBM Corp., SPSS Statistics, Armonk, NY, USA).

## 3. Results

### 3.1. Patients’ characteristics and efficacy of treatments

One hundred-ten female patients with CPP were included in the study: 48 (43.6%) were treated with leuprolide and 62 (56.4%) with triptorelin. Patients treated with triptorelin had higher LH and LH peak levels than leuprolide group at baseline, whereas no other significant difference was observed in the characteristic of patients between the two experimental groups before the beginning of the study (Table 1). At the end of the survey, 41 (85.4%) and 23 (37.1%) of Leuprolide and Triptorelin treated patients reached the bone age maturation and stopped the treatment. No significant difference was observed between patient groups in age, weight, height, PAH, and bone age at the end of treatment (Table 2). Moreover, no significant difference was detected in the absolute change of the same parameters (Table 2). On the other hand, the leuprolide treatment lasted 971 days [790–1,171 days] while the duration of triptorelin administration was 792 days [760–1,003 days] (*p* < 0.001).

**Table 1:** Characteristics of patients at baseline.

	Leuprolide (*N* = 48)	Triptorelin (*N* = 62)	*p*-value
Age (years); [median (Q1–Q3)]	7.5 (7.1–7.8)	7.5 (7.1–7.8)	0.784
Weight (kg); [median (Q1–Q3)]	27.8 (24.0–30.8)	28.7 (24.8–32.7)	0.273
Height (cm); [median (Q1–Q3)]	127.9 (122.8–132.8)	127.5 (121.9–132.1)	0.607
PAH (cm); [median (Q1–Q3)]	150.3 (145.6–154.7)	149.4 (145.8–155.4)	0.938
Bone age (years); [median (Q1–Q3)]	9.6 (9.0–10.3)	9.4 (8.6–10.5)	0.971
FSH baseline (mUI/ml)	2.85 (1.67–3.77)	3.11 (2.00–5.27)	0.263
FSH peak (mUI/ml)	14.1 (11.0–18.4)	13.3 (10.9–15.8)	0.237
LH baseline (mUI/ml)	0.22 (0.10–0.44)	0.35 (0.30–1.42)	<0.001
LH peak (mUI/ml)	6.0 (4.0–13.3)	10.6 (6.5–23.3)	0.006
E2 (pg/ml)	12.0 (5.0–19.5)	7.5 (5.0–18.6)	0.413
SMC (ng/ml)	277.0 (226.0–352.5)	263.5 (205.5–335.5)	0.518
Uterus Longitudinal diameter (mm)	36.5 (32.0–43.0)	38.7 (34.0–43.0)	0.381
Ovarian right (cc)	1.9 (1.2–3.4)	2.0 (1.5–3)	0.455
Ovarian left (cc)	2.0 (1.2–3.3)	1.8 (1.5–2.6)	0.677

**Table 2:** Characteristics of patients at the end of treatment.

	Leuprolide (*N* = 41)	Triptorelin (*N* = 23)	*p*-value
Values at the end of treatment			
Age (years); [median (Q1–Q3)]	10.2 (9.8–10.4)	10.1 (9.8–10.3)	0.353
Weight (kg); [median (Q1–Q3)]	41.5 (35.5–43.8)	40.6 (35.0–46.3)	0.690
Height (cm); [median (Q1–Q3)]	145.7 (138.0–148.6)	142.8 (135.1–149.6)	0.298
PAH (cm); [median (Q1–Q3)]	156.5 (153.7–160.7)	158.2 (151.8–163.7)	0.705
Bone age (years); [median (Q1–Q3)]	11.6 (10.9–12.0)	12.3 (11.0–12.5)	0.077
Duration of therapy (days) [median (Q1–Q3)]	971 (790–1,171)	792 (760–1,003)	0.001
Pubic hair; [*N* (%)]			
Ph 1	13 (31.7%)	7 (30.4%)	0.554
Ph 2	23 (56.1%)	15 (65.2%)	
Ph 3	5 (12.2%)	1 (4.3%)	
Breast growth; [*N* (%)]			
B1	5 (12.2%)	0	0.206
B2	25 (61.0%)	15 (65.2%)	
B3	11 (26.8%)	8 (34.8%)	
Absolute change			
Weight (kg); [median (Q1–Q3)]	12.0 (9.0–15.7)	10.5 (7.8–14.0)	0.203
Height (cm); [median (Q1–Q3)]	15.6 (12.7–16.8)	16.1 (12.0–19.4)	0.542
PAH (cm); [median (Q1–Q3)]	5.3 (1.4–9.9)	4.2 (3.5–9.8)	0.908
Bone age (years); [median (Q1–Q3)]	2.0 (1.4–2.5)	1.8 (1.5–2.0)	0.308

### 3.2. Evaluation of leuprolide and triptorelin tolerability

Side effects were observed in 46 (41.8%) patients. In particular, 19 out of 48 (39.6%) of leuprolide patients and 27 out of 62 (43.6%) triptorelin recipients reported side effects. No significant difference was observed in the number of the reported side effects between the two groups (*p* = 0.558); number of occurred side effects are reported in Table 3.

**Table 3:** Patients and number of symptoms during period study stratified by treatment.

*N*. symptoms	Leuprolide *N* = 48 (%)	Triptorelin *N* = 62 (%)	Total *N* = 110 (%)
0	29 (60.4)	35 (56.4)	64 (58.2)
1	9 (18.8)	18 (29.0)	27 (24.5)
2	5 (10.4)	5 (8.1)	10 (9.1)
≥3	5 (10.4)	4 (6.5)	9 (8.2)

The most frequent side effects were: headache (*N* = 25, 22.7%), increased appetite (*N* = 17, 15.5%) and mood swings (*N* = 16, 14.5%). However, no significant difference was reached between the two groups of treatment (Table 4). Furthermore, all side effects were mild and lasted no longer than 2 weeks, recovered with “ad hoc” symptomatic therapy or disappeared spontaneously. Furthermore, no patient drop-out was recorded and no patients discontinued therapy until the end of survey.

**Table 4:** Side effects observed in the study patients: leuprolide vs. triptorelin.

	Leuprolide *N* = 48(%)	Triptorelin *N* = 62(%)	*p* value	Total *N* = 100 (%)
Headache	8 (16.7%)	17 (27.4%)	0.182	25 (22.7%)
Increased appetite	9 (18.8%)	8 (12.9%)	0.400	17 (15.5%)
Mood swings	8 (16.7%)	8 (12.9%)	0.579	16 (14.5%)
Nausea	5 (10.4%)	1 (1.6%)	/	6 (5.5%)
Pain at the injection site	1 (2.1%)	3 (4.8%)	/	4 (3.6%)
Vomiting	1 (2.1%)	1 (1.6%)	/	2 (1.8%)
Other symtoms	5 (10.4%)	4 (6.5%)	0.452	9 (8.2%)

other symptoms: decreased appetite, articular pain, vaginal discharge, irritability.

No relationship was observed between side effects occurrence and time of treatment, weight, height, chronological age, hormonal levels, as well other radiological clinical parameters, at the beginning of the study (Table 5). Conversely, there was a negative correlation between the risk of side effects with bone age at treatment initiation (*p* = 0.038).

**Table 5:** Factors related to the occurrence of at least one side effects.

	Crude OR (IC 95%)	*p-*value	Adjusted OR (IC 95%)	*p*-value
Leuprolide treatment	0.80 (0.37–1.71)	0.558	1.79 (0.74–4.36)	0.199
Days treatment	1.00 (1.00–1.00)	0.069	1.00 (1.00–1.00)	0.113
Weight (kg)	1.01 (0.95–1.08)	0.772		
Height (cm)	0.97 (0.92–1.02)	0.223		
Chronological age	0.84 (0.51–1.51)	0.517		
Initial bone age	0.67 (0.46–0.98)	0.038	0.67 (0.46–0.98)	0.038
Hormones				
FSH (mUI/ml)	1.06 (0.87–1.29)	0.566		
LH (mUI/ml)	0.83 (0.60–1.14)	0.246		
FSH peak (mUI/ml)	0.99 (0.92–1.07)	0.881		
LH peak (mUI/ml)	0.99 (0.97–1.01)	0.387		
E2 (pg/ml)	1.01 (0.99–1.03)	0.462		
SMC (ng/ml)	1.00 (0.99–1.00)	0.306		
Longitudinal diameter (mm)	0.98 (0.93–1.03)	0.376		
Uterine body/cervix (mm)	1.38 (0.67–2.81)	0.383		
Endometrial (mm)	1.58 (0.70–3.53)	0.268		
Ovarian volume (cc)	1.12 (0.93–1.37)	0.238		

The efficacy/tolerability profile of drugs was evaluated as the ratio between the height or the duration of treatment and the number of observed side effects. No significant difference was observed in these surrogate parameters between leuprolide and triptorelin (Table 6).

**Table 6:** Ratio between duration of therapy or height (both evaluated as surrogate endpoints of efficacy) and number of side effects.

	Leuprolide	Triptorelin	*p*-value
Ratio			
Duration of treatment/*N* side effects; [median (Q1–Q3)]	563 (411–836)	730 (395–914)	0.549
Height at the end of treatment (cm)/*N* side effects; [median (Q1–Q3)]	103.8 (46.5−147.2)	140.2 (133.6–149.7)	0.374

## 4. Discussion

The increase in CPP occurrence observed in the last years may be related to the development of living standards and improvement of quality of life. Food consumption, hormones contained in food as well as the increased adiposity in childhood may contribute to increasing incidence of earlier signs of puberty (23). CPP can lead to premature growth plate closure and subsequent short stature and may increase risk of psychological problems, such as early sexual behavior and psychosexual disorders (24, 25).

GnRHa are mainly prescribed to treat endometriosis, hysteromyoma, prostate cancer, central precocious puberty, and other disorders (26, 27). GnRHa have been widely used since the 1980s in children with rapidly progressing CPP with the aim to restore normal growth, perhaps compromised by the premature closure of bone growth plates, due to sex-hormone impairment (28). Triptorelin is 35 times more effective and has a longer half-life than GnRH. Leuprolide has high affinity with GnRH receptors, decreases gonadotropins release, but also modulates the sensitivity of the ovaries and testes to gonadotropins. Several studies showed that GnRHa treatment is effective in increasing final height when started prior to 6 years of age (29–32). Accordingly, in our study, an increase in predicted adult height was achieved in all patients after treatment with both GnRHa, despite being started at about 7.5 years. No significant difference between the two GnRHa treatments were observed in restoring the growth potential. Moreover, no significant difference was shown between the two patient groups in weight, height, PAH and bone age when the absolute changes were considered at the end of the treatment, despite the duration of treatment resulted significantly lower in triptorelin users, probably due to less time needed to achieve bone age maturation compared to leuprolide group. GnRHa are generally safe (33, 34); however, side effects related to their use were described (35). Pain at the injection site is one of the most common side effects, followed by short term side effects like headache, mood swings, nausea, appetite increase, vomiting that can be classified as mild menopausal symptoms (9, 28, 36). In our study about half of patients (41.8%) reported at least one side effect. Headache, appetite increase, and mood swings were the main side effects observed with a slight and not significant difference between the two groups. Increased appetite was mainly detected in leuprolide treated patients while headache was prevalent in triptorelin group. These results were in accordance with previous studies that reported the same adverse events in pediatric population (28). Moreover, the ratio between the height at the end of treatment or the duration of treatment and the number of observed side effect, both evaluated as readouts of the treatment efficacy were investigated to explore the risk (in terms of side effects appearance) benefit (in terms of efficacy) balance of the two GnRH analogues. The result of these surrogate parameters confirmed the lack of any significant difference between leuprolide and triptorelin.

In this retrospective study, clinical-auxological, bio-humoral and instrumental parameters as well as prescribed drugs were evaluated to identify the factors potentially associated with the onset of GnRHa related side effects. No relationship between side effects occurrence and days of treatment, weight, height, chronological age at diagnosis, hormonal levels, ovarian volume as well as other radiological clinical parameters was observed. By contrast, the probability of side effects appearance significantly reduces with the increase of the initial bone age. As far as we know, no previous study focused attention on the factors that could be related to the onset of side effects. Nevertheless, the current study has some limitations: for instance, it is a single-center study, the number of patients enrolled is relatively small and not all enrolled patients reached the bone maturation and stopped the treatment at the end of the study.

In conclusion leuprolide and triptorelin treatment appear to be effective and safe without significant difference between the two drugs in term of efficacy and tolerability, making both good options for treating CPP.

## Data Availability

The raw data supporting the conclusions of this article will be made available by the authors, without undue reservation.
